# Glial phagocytosis for synapse and toxic proteins in neurodegenerative diseases

**DOI:** 10.1186/s13024-025-00870-9

**Published:** 2025-07-09

**Authors:** Yeseong Choi, Won-Suk Chung

**Affiliations:** 1 Department of Biological Sciences, Korea Advanced Institute of Science and Technology (KAIST), Daejeon, Republic of Korea; 2 Center for Vascular Research, Institute for Basic Science (IBS), Daejeon, Republic of Korea

## Abstract

Glia, as resident immune and supportive cells of the central nervous system, play a critical role in maintaining brain homeostasis. One of their key homeostatic functions is phagocytic capacity in pruning synapses and removing cellular debris/protein aggregates, a process vital for synaptic plasticity and brain maintenance. However, these phagocytic functions are often dysregulated with aging and in neurodegenerative diseases (NDs), such as Alzheimer’s disease, Parkinson’s disease, Huntington’s disease, amyotrophic lateral sclerosis, and frontotemporal dementia. This review aims to examine the phagocytic roles of glia under both physiological and pathological conditions, with a special focus on their interactions with misfolded protein aggregates, including amyloid beta, tau, alpha synuclein, prion, huntingtin, and TAR DNA-binding protein 43. We also explore the fate of ingested molecules after being phagocytosed by glia—whether they are degraded, accumulate intracellularly, or are transferred between cells—and their implications for disease progression. Finally, we review current therapeutic strategies and the potential approaches for modulating glial phagocytosis to mitigate several NDs. We believe that understanding the exact mechanisms of glial phagocytosis and clearance will serve as key elements in developing future treatments for NDs.

## Background

Neurodegenerative diseases (NDs) are a heterogeneous group of disorders characterized by progressive loss of synapses and neurons in the central nervous system (CNS). These diseases include Alzheimer’s disease (AD), Parkinson’s disease (PD), Huntington’s disease (HD), amyotrophic lateral sclerosis (ALS), and frontotemporal dementia (FTD), all of which can lead to a decline in cognitive and/or motor function. A hallmark of many NDs is the accumulation of protein aggregates, such as amyloid beta (Aβ), tau, alpha-synuclein (αSyn), and others.

Traditionally recognized for their supportive roles in the CNS, glia—specifically microglia, astrocytes, and oligodendrocyte precursor cells (OPCs)—are now understood to be key contributors to the pathophysiology of NDs. Recent insights highlight their active roles in maintaining brain homeostasis and facilitating synaptic plasticity for neuronal health and function. In particular, their phagocytic activities, including synapse pruning and the clearance of cellular debris and protein aggregates, are crucial for mitigating the initial pathology of neurodegeneration. However, aging and disease progression often disrupt the balance of glial phagocytosis, leading to abnormal synapse elimination and the accumulation of neurotoxic substrates.

This review aims to elucidate the phagocytic roles of glia in normal aging as well as NDs. We summarize the mechanisms of glial phagocytosis under healthy and pathological conditions and discuss their implications in disease progression. Additionally, we explore the current understanding of the fate of ingested materials following glial phagocytosis. Finally, we review potential strategies for harnessing glial phagocytic activity in therapeutic applications.

## Synaptic pruning and cellular debris clearance

### Glial phagocytosis in development and brain maintenance

The proper phagocytic clearance of cellular debris and synapses by glia is essential for normal brain development and the maintenance of homeostasis [1–5]. Microglia, as professional phagocytes in the brain, rapidly recognize and phagocytose apoptotic cells and cellular debris [6] via specific receptors, including triggering receptor expressed on myeloid cells 2 (TREM2) [7], purinergic receptors [8], Toll-like receptors (TLRs) [9], and TAM (TYRO3, AXL, and MERTK) receptor protein tyrosine kinases [10–12] thereby maintaining homeostasis and promoting tissue repair in the CNS. These receptors are also involved in mediating synapse engulfment by microglia [13–16]. Additionally, components of the complement pathway have been identified as important regulators of synapse phagocytosis by microglia [17–20]. C1q, the initiating protein of the classical complement cascade, binds to synapses marked for removal, facilitating the opsonization of downstream complement proteins such as C3 [17]. Microglial C3 receptors (CR3) then recognize C3-opsonized synapses and mediate their phagocytosis [18]. Altered microglial synapse phagocytosis has been shown to affect synaptic density and mouse behavior [19, 20].

Beyond the complement system, additional molecules regulate synapse refinement by acting as ‘Eat me’ or ‘Don’t eat me’ signals to glia. ‘Eat me’ signals promote the removal of unnecessary synapses, whereas ‘Don’t eat me’ signals prevent excessive synapse elimination. Phosphatidylserine (PS), a well-known ‘Eat me’ signal, can be externalized from certain synapses [21], subsequently tagging them for recognition by several molecules, such as C1q or ADGRG1/GPR56, facilitating microglial engulfment [22, 23]. MERTK, a member of the TAM receptor kinase family, also mediates microglial phagocytosis of PS-positive synapses with the assistance of GAS6, a bridging molecule that links PS to TAM receptors [16]. Conversely, CD47 serves as a ‘Don’t eat me’ signal by interacting with its receptor, SIRPα, to reduce the elimination of active synapses [24]. Another ‘Don’t eat me’ signal, SRPX2, inhibits C1q activity by binding to it. *Srpx2* knockout (KO) enhances C3 deposition and increases the phagocytic capacity of microglia, leading to reduced synapse density [25, 26]. These signaling pathways collectively balance glial synapse pruning to maintain proper neural circuit function.

Astrocytes are also capable of engulfing apoptotic cells, including neurons, microglia, and other astrocytes [27–29]. Microglia and astrocytes exhibit spatiotemporally distinct patterns in their phagocytosis of apoptotic neurons. Microglia primarily target the soma and proximal dendrites, whereas astrocytes preferentially engulf smaller, distal dendrites [12]. Although astrocytes may be less efficient at ingesting whole apoptotic cells compared to microglia [30], they can clear apoptotic cells when microglial phagocytosis is impaired [12, 27]. Furthermore, the phagocytic roles of astrocytes have begun to receive increasing attention since recent findings showed that mice lacking microglia exhibit normal synaptic remodeling, maturation and behaviors [31, 32].

Astrocytes recognize cellular debris through various receptors, including brain-specific angiogenesis inhibitor-1 (BAI1) [33], MEGF10 [34], AXL, and MERTK [27]. Other receptors, such as Fc receptors [35] and scavenger receptors [36], may also be involved in astrocytic phagocytosis. Notably, the phagocytic activity of astrocytes can be influenced by mechanical factors. Astrocytes exposed to shear stress initiate phagocytosis more rapidly than those in static conditions, regardless of fluid flow direction [29].

In addition to apoptotic cells, astrocytes can phagocytose synapses via MEGF10 and MERTK receptors [3, 4]. Interestingly, MEGF10-dependent synapse phagocytosis plays a critical role in mediating synaptic plasticity during learning and memory formation within adult hippocampal circuits [4]. Astrocyte-specific deletion of MEGF10 leads to the accumulation of dysfunctional synapses, resulting in deficits in synaptic plasticity and impaired memory formation. Despite its importance, the synaptic ligands that interact with astrocytic phagocytosis receptors remain unclear. MEGF10 has been shown to bind to C1q [34], but C1q depletion does not appear to affect astrocytic engulfment of apoptotic cells and synapses [28]. This raises the possibility that molecules other than C1q may serve as ligands in MEGF10-dependent synapse refinement.

OPCs give rise to oligodendrocytes, which wrap around axons by forming a myelin sheath. Recently, OPCs have been shown to possess phagocytic capacity in specific brain regions and engage in the engulfment of synapses and other neuronal materials. For example, during the postnatal developmental periods, OPCs in the visual cortex engulf axonal fragments, forming numerous phagolysosomes for the degradation of engulfed materials [37]. Additionally, OPCs internalize and degrade thalamocortical synaptic inputs, and this process is enhanced in response to sensory experience [5]. In contrast, mature oligodendrocytes have a limited phagocytic role compared to OPCs [5], indicating the unique phagocytic ability of OPCs within the oligodendroglial lineage. Further research is needed to investigate the mechanisms and physiological roles of OPCs in synapse remodeling within specific circuits.

Notably, in the developing visual cortex, synapse pruning by OPCs surpasses that by microglia [5]. Similarly, compared to microglia and OPCs, astrocytes play a major role in the phagocytosis of excitatory and inhibitory synapses in the adult hippocampus [4]. These findings suggest that different glia guide synaptic phagocytosis depending on the brain region and the timing of synapse refinement.

### Glial phagocytosis in aging and NDs

Aging affects the phagocytic function of glia, influencing their role in maintaining brain homeostasis. In aged mouse and human brains, C1q and C3, key components of the classical complement pathway, accumulate at high levels, particularly in association with synapses [38, 39]. Given microglia’s role in synapse elimination via the complement pathway, this upregulation of C1q may contribute to enhanced microglial phagocytosis of synapses in aging human brains [40].

In aging mouse and human brains, microglia abnormally accumulate lipid droplets, forming lipid-droplet-accumulating microglia (LDAM) [41]. An increased number of lysosomes are observed near lipid droplets in LDAM. Interestingly, pharmacological inhibition of lipid droplet formation restores the impaired phagocytic capacity of LDAM, indicating that lipid droplet deposition negatively affects their phagocytic function [41]. LDAM are also prominent in progranulin-depleted mice, a model for FTD, where synaptic and lipid droplet accumulation in microglial lysosomes is observed, accompanied by accelerated C1q deposition [42].

Astrocytes also exhibit age-related impairments in phagocytic function. Autophagy-dysregulated astrocytes (APDAs) accumulate autophagosomes in their swollen processes, which colocalize with endosomes, lysosomes, and ubiquitin [43]. Notably, molecular machinery related to synaptogenesis and synapse phagocytosis is mislocalized within these autophagosomes, disrupting the proper regulation of synapse homeostasis. Similar to LDAM, APDAs are also observed in disease mouse models [43]. The appearance of APDAs is significantly accelerated in the hippocampus of AD model mice.

Taken together, aging induces substantial changes in the homeostatic functions of glia, including altered phagocytic activity, which contributes to defective synapse regulation by glia. These functional alterations may be associated with the effects of cellular senescence on glial identity [44, 45]. For instance, hippocampal microglia lose their region-specific characteristics with aging [46], which may contribute to the vulnerability of specific brain regions to neurodegenerative processes.

In the brains of various NDs, both microglia and astrocytes excessively engulf synapses even before neuropathological features appear, contributing to significant synaptic loss [47–51]. This aberrant synapse elimination appears to be closely associated with the activation of the classical complement pathway in response to neurodegenerative processes. In AD mouse models and patient brains, complement components such as C1q and C3 mark synapses and cellular debris for phagocytic removal [47, 52–54]. Similar to their role during development, microglia engage in oligomeric Aβ-induced complement tagging and subsequent synapse phagocytosis [52, 55]. Importantly, it has also been shown that the ablation of C1q, C3, or microglial CR3 preserves synapse density and cognitive function, preventing neuronal degeneration and brain atrophy in AD [52–54, 56]. Interestingly, while astrocytic synapse pruning during development occurs independently of C1q [3], in AD mouse models, astrocytes have been shown to mediate synapse phagocytosis in a C1q-dependent manner, where astrocyte-synapse associations are elevated and correlate with the proportion of C1q-labeled presynapses [54].

The complement pathway also mediates synapse pruning in other NDs. In HD patients and mouse models, elevated C1q and C3 selectively bind to corticostriatal synapses, triggering their engulfment by microglia [57]. Blocking microglial recognition of complement-tagged synapses, either with a C1q-blocking antibody or through CR3 depletion, effectively attenuates synaptic loss and cognitive deficits in HD mice. Similarly, in multiple sclerosis (MS) animal models and patients, presynaptic elements are tagged with C3, resulting in excessive synapse engulfment by microglia [58]. Disrupting the C3-synapse interaction through C3 inhibition prevents microglial synapse engulfment, mitigating synapse loss caused by excessive phagocytosis. Additionally, in PD, C1q opsonizes extracellular neuromelanin deposits in the brain parenchyma, which arise from the progressive degeneration of neuromelanin-containing dopaminergic neurons, leading to their phagocytosis by microglia [59].

How certain synapses in ND brains become susceptible to phagocytic elimination by glia remains unclear. In diseased brains, it has been suggested that persistent calcium accumulation in synapses precedes their removal by microglial phagocytosis. In an MS mouse model, such calcium deposition has also been observed to initiate synapse removal by microglia [60]. In AD brains, toxic molecules such as Aβ oligomers may trigger neuronal hyperactivity and calcium influx at synapses, inducing the exposure of synaptic ‘Eat-me’ signals such as PS [61]. Subsequently, as functional phagocytic machinery in ND-associated glia, the complement pathway [61] or TREM2 [22] mediate the engulfment of PS-positive synapses by microglia. Similarly, blocking the astrocyte-binding domain of MFG-E8, a bridging molecule between PS and integrin receptors, reduces the phagocytosis of AD synaptosomes by astrocytes [62]. Thus, current evidence suggests a model in which localized calcium accumulation in disease states may drive the expression of ‘Eat-me’ signals on synapses, facilitating synapse pruning by glia and contributing to synapse loss.

Although several studies agree that the phagocytic ability of astrocytes is altered in NDs, debate persists over whether it is enhanced or diminished. Different apolipoprotein E (APOE) isoforms distinctly affect the phagocytic capacity of astrocytes. The AD-protective allele *APOE* ε2 enhances astrocytic phagocytosis, whereas *APOE* ε4, the detrimental allele, reduces it [63]. Inflammatory reactive astrocytes downregulate genes encoding phagocytic receptors and endolysosomal proteins, impairing their ability to engulf and clear synaptosomes and cellular debris [64], while promoting the release of endolysosomal cargos [65]. Similarly, Aβ [66] and protofibril αSyn [67] reduce astrocytic capacity to engulf and degrade synapses by downregulating phagocytic receptor expression. Conversely, other studies report increased astrocytic phagocytosis in AD patients [50] and in mouse models expressing Aβ [68] or tau [54] compared to controls. Additionally, astrocytes compensate for impaired microglial phagocytosis in *Trem2* KO models [54]. These discrepancies suggest that factors such as experimental models, the specificity of phagocytic targets and the stage of disease progression may all influence the phagocytic function of astrocytes.

In the spinal cord of familiar ALS patients, misfolded superoxide dismutase 1 (SOD1) has been detected in astrocytes, microglia, and oligodendrocytes [69]. However, the role of oligodendrocytic SOD1 in neurodegeneration remains controversial [70, 71]. Similarly, while microglia expressing mutant SOD1 contribute to motor neuron degeneration in ALS by releasing neurotoxic nitric oxide and reactive oxygen species [72–74], the phagocytic role of microglia in SOD1-related pathology has yet to be investigated. Astrocytes carrying mutant SOD1 play a particularly detrimental role in the late stages of ALS [75, 76]. They regulate microglial activation and nitric oxide synthesis, thereby contributing to neuronal death and behavioral deficits. These deleterious effects may be associated with increased phagocytic and lysosomal activity observed in mutant SOD1-expressing astrocytes [77]. At late-stage ALS, astrocytes exhibit enrichment of genes related not only to immune responses but also to phagocytosis and lysosomal pathways. Further studies are required to determine whether the enhanced phagocytic activity of astrocytes is a cause or consequence of disease progression.

## Glial phagocytosis for protein aggregate clearance

### Amyloid beta

The inappropriate cleavage of amyloid precursor protein (APP) generates Aβ. An imbalance between Aβ production and clearance leads to its aggregation and deposition in the brain as plaques, one of the earliest neuropathological hallmarks of AD. These plaques trigger neuroinflammation and contribute to neurodegeneration, ultimately resulting in cognitive decline and other symptoms characteristic of AD.

Glia are closely associated with Aβ plaques, and astrocytes and microglia are recruited through partially distinct but overlapping mechanisms. Astrocytes have been shown to form multicellular aggregates around Aβ plaques, driven in part by interactions between APOE and low-density lipoprotein receptor-related protein 1 (LRP1) [78]. These interactions not only promote astrocyte recruitment to plaques but are also implicated in subsequent Aβ dissolution processes [78], as described below. Meanwhile, microglia are rapidly recruited to Aβ plaques through the recognition of Aβ itself or associated signals via diverse receptors, including TAM receptors [79], TREM2 [80, 81], PIEZO1 [82], and chemokine-like receptor 1 (CMKLR1) [83]. Microglia surrounding Aβ deposits appear to limit plaque expansion. Microglial depletion throughout the brain in an AD mouse model results in an increase in both the number and size of Aβ plaques, which also display a more diffuse morphology [55]. Notably, certain recruitment pathways may be shared between astrocytes and microglia, since both cell types express receptors such as TAM receptors [3, 27, 84] and LRP1 [85] that facilitate Aβ plaque association.

**Fig. 1:**
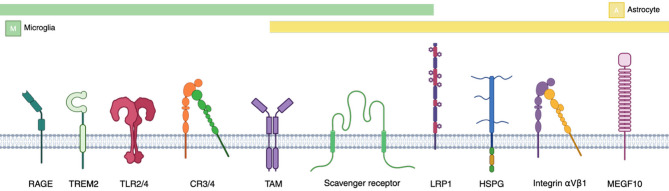
Schematic representation of membrane receptors expressed in microglia and astrocytes involved in the uptake of aggregation-prone proteins. Receptors are aligned by cell-type specificity, with green and yellow bars indicating microglia and astrocyte expression, respectively. Bars spanning both regions represent receptors expressed in or possibly shared by both cell types. The detailed uptake mechanisms and associated receptors are summarized in Table 1

Research has demonstrated that microglia and astrocytes are involved in Aβ uptake, showing a preferential affinity for oligomeric forms over fibrils [86–90]. Extensive efforts have been made to identify the molecular mechanisms underlying Aβ uptake by microglia and astrocytes. The major receptors and associated pathways mediating the uptake of Aβ and other protein aggregates is presented in Fig. 1; Table 1. A cell surface receptor complex, including the B-class scavenger receptor CD36, α6β1 integrin, and CD47, has been identified as a mediator of the interaction between Aβ and microglia [91, 92]. Furthermore, blocking these receptors significantly reduces the phagocytic capacity of astrocytes [93]. However, these receptors also recognize other protein fibrils, indicating that they do not constitute a receptor complex specific to Aβ. Scavenger receptor A (SR-A) can also mediate Aβ uptake by microglia and astrocytes, although the detailed molecular mechanisms may differ. Activated SR-A promotes the uptake of both monomeric and oligomeric Aβ by astrocytes, whereas it facilitates only oligomeric Aβ uptake by microglia [94]. This suggests that astrocytes and microglia phagocytose Aβ in distinct ways depending on its structural form.

**Table 1:** Cell type-specific receptors and uptake mechanisms involved in the internalization of aggregation-prone proteins by glial cells. This table summarizes the known receptors and pathways utilized by microglia, astrocytes, and OPCs/oligodendrocytes for the uptake of aggregation-prone proteins involved in neurodegenerative diseases. Not all mechanisms are equally established across all glial types. Some mechanisms are inferred based on homologous pathways or indirect evidence

Protein	Microglia	Astrocytes	OPCs / Oligodendrocytes
Amyloid β	Scavenger receptors (CD36 [90, 91], SR-A [93]); TAM receptors (AXL, MERTK) [78]; TREM2 [14, 79]; TLR2/4 [97–99]; CR3 [100]; PIEZO1 [81, 102]	Scavenger receptors (CD36 [92], SR-A [93]); LRP1 [95]; APOE-LRP1 interaction [84, 96]; MEGF10 [87]	Macropinocytosis [111] / Not clearly established
Tau	CR4 [130]; CX3CR1 [131]; Synapse pruning [50, 54]	HSPG-mediated uptake [143]; Integrin-dependent endocytosis (αV/β1) [148]; Synapse pruning [50, 54, 65]	Not established
α-Synuclein	Clathrin-mediated endocytosis [161]; TLR2 [162]; TLR4 [164–166]; RAGE [169]; CD36 [173]	Dynamin-dependent endocytosis [176, 177, 180]; Annexin A2-associated clearance [181]; Clusterin-induced uptake [180]	Dynamin-dependent internalization [183]
PrP^Sc^	Not established	Endocytosis into lysosomes [197–199]	Minimal role [186]
TDP-43	Rarely detected [202]; TREM2 [208]	Rarely detected [202, 204]	Not established
Mutant HTT	Not established	Draper (drosophila homolog of MEGF10)-mediated internalization [211, 212]	Not established

Similar to its role in neurons [95], LRP1 mediates astrocytic uptake and degradation of Aβ. Depletion of LRP1 in astrocytes increases Aβ plaque accumulation without affecting Aβ production [96]. Notably, LRP1 binds to APOE, a protein co-deposited with Aβ in AD mouse brains [97], suggesting that LRP1-APOE interactions play a critical role in both recruitment and clearance functions of astrocytes.

Phagocytic receptors such as TAM receptors and MEGF10 also play key roles in Aβ internalization by microglia and astrocytes. Microglia utilize AXL and MERTK to detect and phagocytose Aβ plaques decorated with PS and GAS6 [79]. In astrocytes, Aβ phosphorylates and activates MEGF10, enhancing its involvement in Aβ phagocytosis [88]. Supporting this finding, MEGF10-overexpressing fibroblasts engulf more Aβ compared to controls, further highlighting the role of MEGF10 in Aβ clearance [88].

TREM2 directly binds to Aβ and facilitates microglial phagocytosis, a function that is diminished in TREM2-deficient primary microglia cultures and mouse brains [14, 80]. Interestingly, AD-associated mutations in TREM2, recognized as significant genetic risk factors for the disease, reduce its binding affinity for Aβ [81]. TLRs, particularly TLR2 and TLR4, also mediate interactions with Aβ aggregates [98–100]. Upon recognizing Aβ, these pattern recognition receptors activate microglia, eliciting a pro-inflammatory response and altering phagocytic activity. Complement components are also implicated in Aβ phagocytosis by microglia. Using C3 and CR3, microglia internalize synthetic fibrillar Aβ42 and transport it to lysosomes for degradation [101]. However, prolonged activation of the C3/CR3 axis has been shown to impair microglial phagocytosis [102].

Microglia also utilize PIEZO1, a mechanotransduction ion channel, to recognize Aβ plaques and suppress their expansion. Among known mechanosensory ion channels, PIEZO1 is the most highly expressed in primary microglia [82]. In AD mouse models and AD patient brains, microglial PIEZO1 expression is elevated in regions associated with Aβ plaques, which exhibit higher tissue stiffness compared to areas without Aβ accumulation [82]. The stiffness of fibrous Aβ serves as a mechanical stimulus, leading to increased expression and activation of PIEZO1. This activation triggers transient Ca²⁺ influx through PIEZO1 channels, enhancing microglial migration and lysosomal activity to facilitate Aβ phagocytosis [103]. In AD mouse models, PIEZO1 depletion in microglia impairs their Aβ phagocytic capacity, resulting in significantly accelerated Aβ plaque accumulation, expansion, and cognitive dysfunction [82].

Under normal conditions, astrocytes do not express PIEZO1. However, in the presence of Aβ plaques, reactive astrocytes in murine AD models and patient brains exhibit increased PIEZO1 expression [104, 105]. In addition, conditioned media from Aβ-exposed microglia has been shown to induce PIEZO1 upregulation in astrocytes [105]. These findings suggest that PIEZO1 activation in astrocytes may be mediated by microglia-derived secreted molecules, potentially enhancing astrocytic responses for sensing and clearing Aβ plaques in AD. Thus, these multifaceted receptor systems underscore the complexity of glial interactions with Aβ in the pathogenesis of AD.

Microglial roles during AD progression are dynamic and stage-dependent. In AD mouse models with Aβ plaques, a subtype of microglia known as disease-associated microglia (DAM) has been identified, in close proximity to plaques [106]. During the early stages of the disease, DAM exhibit downregulation of microglial homeostatic genes. As the disease progresses, however, genes related to lysosomal, phagocytic and immune response pathways become significantly upregulated [106]. This dynamic transition of microglia may explain why microglia appear to participate not only in plaque clearance but also in plaque formation and Aβ propagation in numerous studies [107–110]. A recent study reconciles these seemingly contradictory observations by demonstrating the dual roles of microglia at different stages of the disease [111]. In early stages of the disease, homeostatic microglia promote Aβ plaque seeding by facilitating initial aggregation. At later stages, however, microglia play a protective role by compacting plaques and restricting their expansion [112]. This dynamic shift reflects the context-dependent functions of microglia during disease progression, underscoring the complexity of microglial involvement in AD progression.

The roles of OPCs and mature oligodendrocytes in Aβ plaque formation and clearance remain areas of active investigation. OPCs are frequently observed near plaques in AD mouse brains [112], suggesting their potential role as Aβ phagocytes. In vitro studies demonstrate that OPCs internalize Aβ through macropinocytosis and attempt to degrade it via autophagy-mediated pathways [112]. In contrast, mature oligodendrocytes may contribute to Aβ production by expressing APP-cleaving enzymes [113].

Although microglia-derived signals may influence OPC function, including their capacity to engulf synapses [5], the extent to which OPCs interact with other glia, such as microglia and astrocytes, during Aβ clearance remains poorly understood. Further research is needed to elucidate these complex cellular dynamics in Aβ metabolism and pathology.

### Tau

Tauopathies are heterogeneous NDs characterized by the accumulation of abnormally hyperphosphorylated tau in neurons and glia. Tau is predominantly found in the axons of neurons, where it stabilizes microtubules. Under pathological conditions, hyperphosphorylated tau detaches from microtubules, accumulates in the somatodendritic compartment of neurons, and eventually forms neurofibrillary tangles (NFTs). In the brains of AD patients, tau spreads through synaptically connected neurons, progressing from the entorhinal cortex (EC) to the hippocampus and, finally, to the neocortex [114, 115]. Tau propagation is associated with progressive neurodegeneration, although the underlying mechanisms remain largely unknown.

It has been proposed that toxic tau can be released into the extracellular space spontaneously through neuronal activation, independent of neuronal cell death [116–118], and neighboring neurons can then recognize and take up the secreted tau [119, 120]. While tau can also be secreted by neurons via extracellular vesicles (EVs) [121], less than 1% of EVs from the brains of AD patients contain pathological tau filaments [122]. Similarly, only a small fraction of tau released from N2a cells [118] and tau-overexpressing rat primary neurons is associated with vesicles [121].

Although tau expression is significantly higher in neurons compared to other brain cells [123], tau inclusions are also observed in glia, including astrocytes, microglia, and oligodendrocytes. This observation suggests that glia may internalize tau originating from neurons. Microglia can recognize and engulf exogenous tau both in vitro and in vivo [124, 125]. Primary microglia from human AD patients and tauopathy mouse models consistently contain tau protein, despite lacking its expression at the transcriptional level [126]. In a tau mouse model that overexpresses tau specifically in EC neurons, activated microglia were found to be surrounding phosphorylated tau-containing patches in the axon-rich region of the dentate gyrus [127]. Additionally, microglia may engulf insoluble tau during the phagocytosis of neurons with tau inclusions. In AD patient brains and tau mouse models, microglia are closely associated with NFT-containing neurons [124, 128]. Notably, tau aggregates have been detected in microglia following the phagocytosis of PS-exposing neurons [128], suggesting that tau may be transferred from dying neurons to microglia through cellular phagocytosis.

Several mechanisms have been proposed to explain tau recognition and internalization by microglia. Microglia migrate toward extracellular tau oligomers through P2Y12-associated actin remodeling [129, 130]. In vitro experiments with primary microglia indicate that complement receptor 4 (CR4) mediates the phagocytosis of seed-competent tau aggregates but not monomers [131]. Supporting these findings, the genes *Itgax* and *Itgb2*, which encode the proteins forming CR4, are upregulated in the brains of AD patients and tau AD mouse models [131]. Tau engulfment by microglia can also occur through ligand-receptor interactions between tau and CX3CR1 [132]. Although DAM display the decreased gene expression level of *Cx3cr1* in mouse models with Aβ plaque deposition [106], CX3CR1 expression progressively increases in the brains of individuals with AD as the disease advances [132]. Tau is capable of binding to CX3CR1 on microglia due to its similarity in amino acid sequence to CX3CL1 [132], the natural ligand of CX3CR1. However, disease-associated phosphorylation of tau induces conformational changes, weakening its binding affinity and thereby preventing tau uptake by microglia [132]. Interestingly, several studies suggest that microglia alone are insufficient for the efficient internalization and degradation of tau [133–135]. In the brains of AD patients at various disease stages, both the number of microglia and the area they cover are significantly reduced as AD severity progresses [134]. Moreover, microglia show a marked decline in their phagocytic capacity and adopt a senescence-associated phenotype after engulfing tau-bearing neurons [135]. These findings indicate that the phagocytosis of tau-containing neurons induces toxic effects, contributing to microglial dysfunction.

Astrocytes have been shown to harbor tau inclusions in the brains of patients with various tauopathies and in corresponding mouse models, despite the absence of robust tau transgene expression [127, 136–139]. The accumulation of tau in astrocytes disrupts mitochondrial localization and function, impairing calcium buffering and ATP synthesis [140, 141]. These mitochondrial defects contribute to neuronal network dysfunction and memory impairment [140]. Additionally, when human iPSC-derived astrocytes are exposed to tau fibrils from AD brain extracts, they become reactive and secrete pro-inflammatory cytokines [141]. Conditioned media from these reactive astrocytes induces synaptic dysfunction in human iPSC-derived neurons [141], further emphasizing the detrimental effects of tau accumulation in astrocytes.

How does tau get ingested by astrocytes? Astrocytes form close physical associations with neuronal synapses, known as the tripartite synapse, highlighting their potentially critical role in receiving tau released from synapses. It is well documented that heparan sulfate proteoglycans (HSPGs) mediate tau internalization in neurons [142, 143]. Interestingly, it has been suggested that astrocytes also phagocytose tau protofibrils via an HSPG-associated mechanism [144]. However, for monomeric tau internalization, the involvement of HSPGs appears to depend on conformational differences between tau isoforms [145, 146]. Furthermore, studies indicate that the interaction between HSPGs and tau—unlike with Aβ or αSyn—relies on the size and sulfation patterns of HSPGs [143, 147, 148]. These findings suggest that multiple mechanisms govern tau uptake by astrocytes, depending on its conformation or aggregation state.

Cell adhesion molecules, such as αV/β1 integrin, can directly bind to free-form tau monomers and protofibrils [149]. This integrin complex mediates integrin-dependent endocytosis of filamentous tau by primary astrocytes, inducing a reactive astrocyte state and the release of pro-inflammatory molecules [149]. LRP1 is another potential receptor mediating tau uptake by glia. Neuronal LRP1 facilitates tau uptake through interactions between the microtubule-binding domain and N-terminus of tau and the extracellular domains II and IV of LRP1 [150]. Given that glia with phagocytic properties, including microglia, astrocytes, and OPCs, also abundantly express LRP1 [85], it is plausible that various glia may use LRP1 as a mechanism to uptake tau from neurons.

Despite evidence showing the direct uptake of extracellular tau by glia, it is important to note that the amount of extracellular tau is likely extremely low compared to intracellular neuronal tau in AD [151]. Additionally, extracellular tau is predominantly monomeric [152] or truncated [153], both of which are generally considered non-pathogenic forms, and can be detected even in wild type mice [117, 154]. These observations raise the possibility that pathological tau transfer to glia may occur through mechanisms other than direct extracellular tau uptake. One such mechanism may involve synaptic pruning via glial phagocytosis and the subsequent transfer of synaptic tau from neuron to glia. Indeed, tau is closely associated with synaptic components across various models, including in vitro primary murine neurons [143], flies [155], mice [47, 156], and humans [50, 157], where it has been shown to induce synaptic dysfunction [155, 156, 158]. In the brains of AD patients, pathogenic tau accumulates in both pre- and postsynaptic compartments, even in regions lacking NFTs [50, 157]. Notably, it has been suggested that tau-associated synapses can be eliminated by glia, particularly microglia and astrocytes, both in vitro and in vivo [54, 66]. A recent study demonstrated that microglia and astrocytes actively engulf synapses containing oligomeric tau in human AD brains [50]. The extent of oligomeric tau accumulation in synapses and the degree of tau-containing synapse pruning negatively correlate with cognitive function in patients [50]. Interestingly, tau accumulation in synapses may trigger selective engulfment by glia as a mechanism to eliminate dysfunctional synapses. In the AD brain, approximately 30% of postsynapses and 50% of presynapses are positive for tau oligomers [50]. However, the proportion of oligomeric tau-positive synapses phagocytosed by microglia and astrocytes is nearly double [50], suggesting that these glia preferentially engulf tau-containing abnormal synapses over healthy ones.

Tau accumulation in oligodendrocytes disrupts their function, including the maintenance of myelin integrity [159], and leads to progressive degeneration of both axons and oligodendrocytes [160]. The mechanism underlying tau transfer to oligodendrocytes remains largely unknown. However, studies suggest that oligodendrocytic tau pathology can spread across the mouse brain independently of neuronal tau pathology [137].

### alpha-synuclein

αSyn is an intrinsically disordered protein highly concentrated in neuronal synapses. This protein is implicated in several NDs, collectively termed synucleinopathies, which include PD, dementia with Lewy bodies (DLB), and multiple system atrophy (MSA). The aggregation of αSyn into insoluble fibrils is a key pathological hallmark of these diseases, leading to the formation of Lewy bodies and glial cytoplasmic inclusions. These aggregates contribute to the loss of dopaminergic neurons, motor dysfunction, and disease progression.

Neuronal-derived αSyn has been detected both within and on the surface of EVs, secreted via calcium-dependent exocytosis [161–163]. When injected into the striatum of mice, EVs isolated from the plasma of PD patients propagate αSyn across anatomically connected regions accompanied by microglia activation [164]. However, given that αSyn primarily localizes on the surface of EVs rather than within their lumen [164], it remains plausible that αSyn detached from EVs may act both as an activator of microglia and as a seed for αSyn propagation.

Microglia recognize and internalize αSyn, triggering their activation and altering phagocytic functions [165]. The central non-amyloid-β component domain, which is responsible for the β-sheet formation and aggregation of αSyn, is crucial for its phagocytic elimination by microglia [165]. Several plasma membrane proteins on microglia have been identified as potential counterparts that interact with aggregated αSyn and mediate its internalization [166]. For instance, clathrin, an essential protein involved in endocytosis and vesicular trafficking, colocalizes with ingested αSyn in microglia [166], highlighting the role of endocytosis in microglial αSyn uptake.

In vitro and in vivo studies have demonstrated that neuronal αSyn activates microglia through the TLR2 signaling pathway, inducing the release of pro-inflammatory cytokines [164, 167]. In the brains of DLB patients and αSyn transgenic mice, TLR2 expression is significantly elevated in both neurons [168] and microglia [167]. *Tlr2* KO not only suppresses microglial activation but also reduces αSyn binding to microglia, a process that can also be inhibited by a TLR2-blocking antibody [167]. Interestingly, the interaction between TLR2 and αSyn is influenced by αSyn’s conformation and aggregation state. Compared to monomers, dimers, and fibrils, β-sheet-enriched αSyn oligomers exhibit greater affinity for TLR2 and a stronger capacity to activate microglia [167].

Other studies have highlighted the role of TLR4 in αSyn uptake by microglia. Both monomeric and fibrillized αSyn are internalized through a TLR4-dependent mechanism [169–171]. Notably, TLR4-depleted mouse models exhibit accelerated αSyn propagation and exacerbated neurodegeneration [169, 171]. In contrast, αSyn internalization by astrocytes occurs independently of TLR4 [170, 172]. Interestingly, αSyn does not induce TLR4 endocytosis, distinguishing its mechanism of action from that of the LPS-TLR4 signaling pathway [173]. These findings suggest that while TLR4 is involved in αSyn uptake by microglia, it is likely the downstream TLR4 signaling, rather than TLR4 itself, that modulates αSyn uptake and propagation.

Other cell-surface receptors have been proposed as candidates for αSyn recognition and ingestion by microglia. For instance, depletion of the receptor for advanced glycation end products (RAGE) in primary microglia reduces αSyn binding and subsequent pro-inflammatory cytokine secretion [174]. However, αSyn binding is not entirely abolished in RAGE-depleted microglia [174], suggesting that multiple mechanisms mediate the interaction between αSyn and microglia. TREM2 has been also linked to microglial responses to αSyn. TREM2 depletion or anti-TREM2 antibody treatment reduces microglial phagocytic capacity and downstream responses [175, 176]. However, the absence of a direct interaction between TREM2 and αSyn [176] suggests that TREM2 contributes to microglial phagocytosis through a mechanism not specific to αSyn. Additionally, microglial internalization of αSyn is inhibited by scavenger receptor blockers [177]. An antibody against CD36, a specific scavenger receptor, reduces the interaction between αSyn and primary microglia [178]. Furthermore, *Cd36* KO attenuates αSyn-induced microglial activation [179] and decreases αSyn levels in whole-cell lysates of bone marrow-derived macrophages [180]. While these receptors interact with αSyn and contribute to microglial activation, further studies are required to elucidate their roles in microglial phagocytosis of αSyn and the precise mechanisms underlying these processes.

Similar to microglia, astrocytic accumulation of αSyn has been observed in primary astrocytes [181, 182], astrocytes derived from PD iPSCs [183], mouse brains with neuron-specific αSyn overexpression [181], and the brains of DLB patients [181]. Primary astrocytes can internalize secreted αSyn from the conditioned media of neuronal cells and αSyn aggregates from PD patient extracts, leading to the formation of Lewy body-like inclusions near the nucleus [181, 182, 184]. This uptake process may occur via dynamin-associated endocytosis, with internalized αSyn trafficked to lysosomes through the endo-lysosomal pathway [181, 182, 185]. Other molecular mechanisms mediating astrocytic endocytosis of αSyn have also been suggested. Mutations in LRRK2 suppress the capacity of astrocytes to internalize and clear αSyn [186]. This impairment in lysosomal degradation is associated with downregulated annexin A2 expression [186], a protein involved in endocytosis and phagocytic transport. However, the mechanism by which mutant LRRK2 reduces annexin A2 expression remains unclear. Clusterin, an extracellular chaperone, interacts with αSyn preformed fibrils in the extracellular space, blocking αSyn invasion into astrocytes [185]. Further investigations are needed to clarify the cause and consequences of clusterin-induced reductions in astrocytic αSyn uptake. Similar to tau, LRP1 directly binds to the N-terminus of αSyn, specifically targeting lysine residues, and mediates the uptake of soluble αSyn by neurons [187]. Given the abundant expression of LRP1 in glia, its role in astrocytic αSyn phagocytosis warrants further investigation.

Oligodendrocytes can internalize αSyn monomers and oligomers but rarely fibrils, through a dynamin-dependent mechanism [188]. Recombinant αSyn has been detected within oligodendrocyte cell lines [188, 189], primary oligodendrocytes [189], and oligodendrocytes in the mouse cortex [188] following its administration or injection. Interestingly, transplanted OPCs and oligodendrocytes have been found to contain human αSyn overexpressed in the host rat brain [188]. Similarly, oligodendrocytes differentiated from human neural stem cells can ingest and accumulate αSyn released from neuronal cells [190]. Given that mature oligodendrocytes do not express αSyn, these findings suggest that oligodendrocytes acquire αSyn transferred from neurons, contributing to its accumulation in synucleinopathies such as MSA. A summary of the glial receptors and uptake mechanisms for αSyn, along with those identified for other aggregation-prone proteins, is provided in Fig. 1; Table 1.

### Other proteins

While Aβ, tau, and αSyn are the most well-known proteins involved in NDs, other proteins can also form aggregates and be phagocytosed by glia. Prion diseases, also known as transmissible spongiform encephalopathies, are characterized by the misfolding and aggregation of prion protein (PrP^Sc^). In the brain, the normal cellular prion protein (PrP^C^) is expressed on neurons, astrocytes, and oligodendrocytes. However, the role of oligodendrocytes in disease progression appears to be minimal, as neuronal PrP^Sc^ infection and other disease-related pathologies are absent in a transgenic mouse model where PrP^C^ is expressed exclusively in oligodendrocytes [191]. Microglia phagocytose PrP^Sc^ before engulfing damaged neurons, a process that occurs prior to clinical onset [192]. Microglia isolated from PrP-infected mice exhibit an enhanced capacity for the phagocytosis of synaptic and myelin debris, accompanied by upregulated expression of phagocytosis-associated genes [193]. Microglial depletion increases susceptibility to PrP infection and promotes the accumulation of PrP^Sc^ insoluble aggregates in both ex vivo mouse brain slices [194, 195] and in vivo mouse brains exposed to PrP [195]. Notably, this increase in aggregates is reversed by the introduction of macrophages [194]. Although microglial ablation reduces the levels of pro-inflammatory cytokines elevated by PrP infection, overall prion pathology is exacerbated [195]. These findings suggest that microglia play a neuroprotective role in prion disease, potentially through various mechanisms beyond their inflammatory cytokine production such as enhanced phagocytosis of protein aggregates and damaged cells.

Interestingly, reactive astrocytes may compensate for microglial uptake and degradation of PrP in the context of microglial depletion. In another microglial depletion mouse model, which exhibits reduced disease-associated PrP levels in the brain, reactive astrocytes emerge earlier compared to wild-type mice, even before microglial ablation-dependent changes in PrP accumulation [196]. These reactive astrocytes demonstrate a pronounced capacity to engulf postsynapses, suggesting their potential role in mitigating the impact of microglial depletion. Although astrocyte-specific PrP expression does not induce prion disease pathologies [197], the accumulation of PrP^Sc^ in astrocytes has been consistently demonstrated both in vitro [198] and in vivo [199–201]. Astrocytes actively internalize PrP aggregates through endocytosis [202], primarily sorting them into endo-lysosomal compartments [202–204]. However, the detailed mechanisms underlying this internalization remain to be investigated.

TAR DNA-binding protein 43 (TDP-43) is a nuclear protein that binds to RNA and DNA, regulating gene transcription, RNA splicing, and stability. It is primarily associated with specific NDs, such as ALS and FTD. In ALS and FTD, TDP-43 redistributes to the cytoplasm, where it undergoes cleavage, hyperphosphorylation, and aggregation. TDP-43 deposits have been observed in neurons [205, 206] and oligodendrocytes [207, 208], but are rarely found in astrocytes or microglia [207]. This limited detection in glia may be due to the preferential accumulation of TDP-43 in neurons [206] or the stronger degradation capacity of astrocytes [209]. In iPSC-derived neurons and astrocytes, the seeded aggregation of TDP-43 is enhanced by proteasome inhibition [209]. However, while neurons exhibit increased apoptosis under these conditions, astrocytes remain resistant. Astrocytes are more likely to receive TDP-43 aggregates from neurons without transferring them back, and they exhibit lower vulnerability to toxic TDP-43 oligomers [209]. By efficiently degrading neuronal TDP-43, astrocytes may protect neurons from TDP-43 accumulation and cell death, potentially through the secretion of neuroprotective factors.

Several studies have demonstrated the role of microglia in TDP-43 phagocytosis. During the early recovery phase of TDP-43 proteinopathy, microglia become reactive independently of prior neuronal death and phagocytose neuronally expressed TDP-43 [210]. Microglial depletion during this phase significantly impairs the resolution of disease phenotypes, including TDP-43 deposition, behavioral deficits, and neurodegeneration. In the zebrafish spinal cord, microglia internalize TDP-43 while clearing UV-injured neurons containing TDP-43 [211]. In the absence of microglia, TDP-43 leaks out of the nucleus, potentially due to nuclear pore defects [212], and becomes mislocalized to the cytoplasm and axons, eventually being released into the extracellular space during neuronal degeneration [211]. In the brains of human patients, as well as in vitro and in vivo TDP-43 mouse models, an interaction between extracellular TDP-43 and TREM2 on microglia has been identified [213]. Specifically, the C-terminal residues of TDP-43 bind to TREM2, promoting the transition of microglia into a reactive, disease-associated state that facilitates TDP-43 phagocytosis. TREM2 deficiency prevents this microglial state transition and impairs phagocytosis, resulting in increased TDP-43 accumulation, behavioral deficits, and neuronal loss [213].

HD is an autosomal dominant ND caused by a CAG expansion in the huntingtin gene, leading to the intracellular aggregation of mutant huntingtin protein (HTT), primarily in neurons. HTT deposits have also been detected in glia, including astrocytes, microglia, and oligodendrocytes, in the brains of various mouse models and human patients [214, 215]. However, only a few studies using Drosophila have explored the role of glial phagocytosis in HD progression [216, 217]. In Drosophila, glia internalize mutant HTT from neurons via the phagocytic receptor Draper [216]. This transfer of HTT requires close proximity or direct physical contact between donor neurons and recipient glia. Once internalized, the neuronal mutant HTT interacts with glial wild-type HTT, forming aggregates that associate with endogenous chaperones [216]. Interestingly, Draper and its downstream phagocytic machinery are essential for the aggregation of HTT derived from both neurons and glia [216]. HTT aggregates are primarily found in ensheathing glia, where Draper mediates the clearance of cellular debris in the adult fly brain [217]. Given that MEGF10, the human homolog of Draper, functions as a phagocytic receptor in astrocytes, further investigations in mouse and human models are needed to clarify the role of MEGF10-dependent phagocytosis in HTT propagation between neurons and glia.

## Beyond phagocytosis

**Fig. 2:**
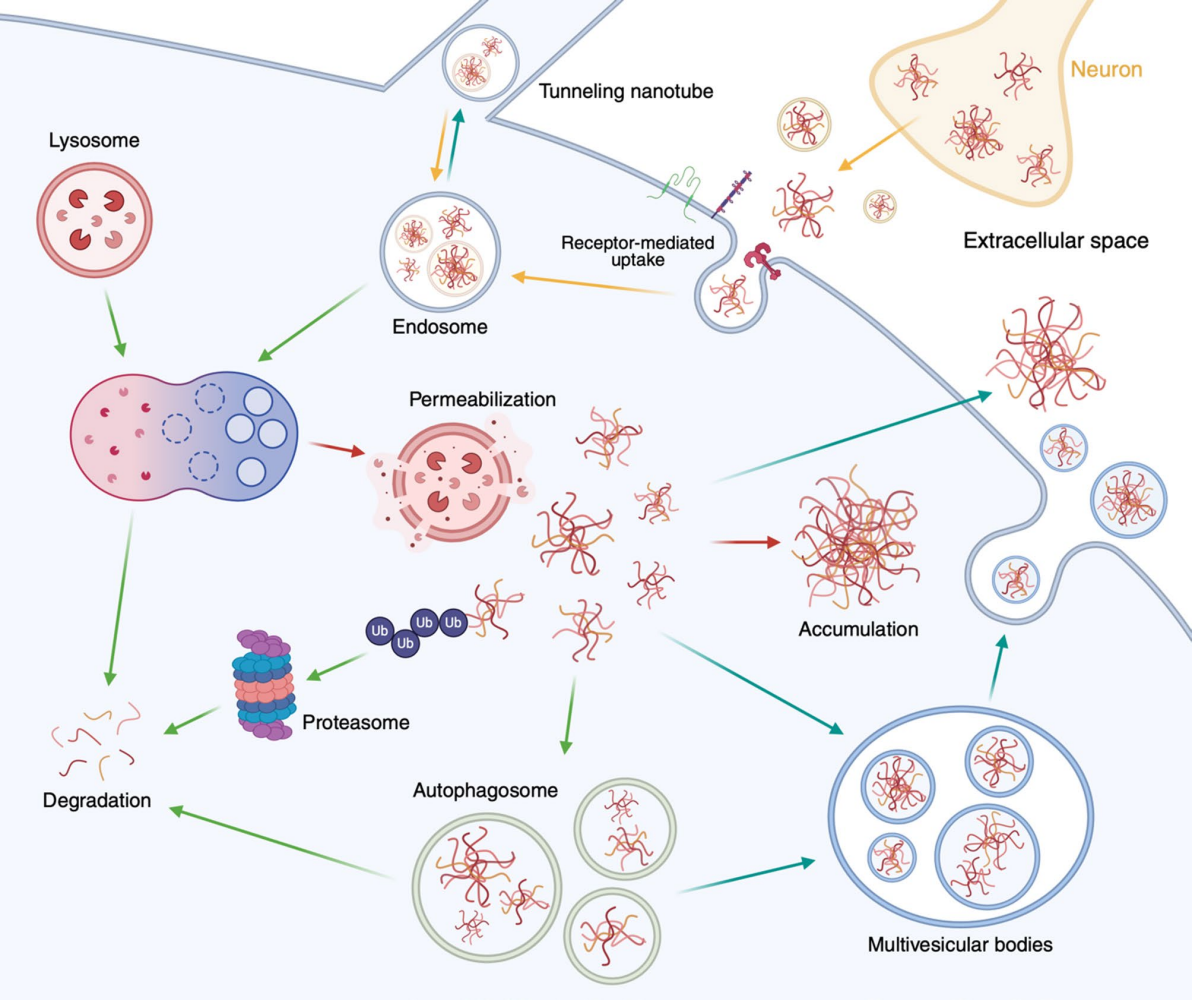
Multiple intracellular pathways of protein aggregates in glia. Protein aggregates internalized by glia can follow multiple intracellular pathways. These internalized proteins are typically trafficked to lysosomes for enzymatic degradation. However, lysosomal dysfunction, such as deacidification or permeabilization, can impair the protein degradation capacity of glia, inducing protein escape into the cytosol. In the cytosol, aggregates are targeted for proteasomal degradation via ubiquitination or sequestered in autophagosomes for autophagy-mediated clearance. Impairments in these pathways can lead to protein accumulation within the cells and/or the re-release of partially degraded proteins. Glia can further contribute to the pathogenic protein dissemination by releasing seed-competent aggregates into the extracellular space through multivesicular bodies or direct cell-to-cell transfer via tunneling nanotubes. These processes highlight the dual roles of glia in the clearance and propagation of pathogenic protein aggregates. Arrows are color-coded to indicate the direction of distinct trafficking routes: yellow for uptake into glia, green for degradation, red for cytosolic accumulation and blue for release from glia

Once glia internalize protein aggregates, they attempt to degrade the ingested molecules (Fig. 2). In vitro studies have shown that both microglia and astrocytes can internalize and degrade Aβ [218] and tau [125], as evidenced by the reduced concentrations of these proteins in culture media and cell lysates after treatment. The degradation of internalized protein aggregates by glia is closely linked to lysosomal function. RNA sequencing data from AD mouse models reveal that genes associated with lysosomal function are significantly enriched in disease-associated microglia [106] and astrocytes [219], suggesting an effort to enhance lysosomal degradation of toxic proteins. Indeed, an early study observed that Aβ internalized by microglia is trafficked to acidic endosomes and lysosomes [220]. Additionally, increasing lysosomal biosynthesis by enhancing the expression and activity of transcription factor EB (TFEB), a master regulator of autophagy and lysosomal biogenesis, promotes Aβ plaque clearance by both astrocytes [221] and microglia [222]. Similarly, TFEB overexpression in astrocytes enhances phosphorylated tau uptake and degradation, attenuating tau propagation in PS19 mouse models [145]. Sirtuin1, a protein associated with aging and longevity, also boosts Aβ degradation by regulating lysosomal number and function [223]. Likewise, PrP fibrils internalized via endocytosis are trafficked to late endosomes and lysosomes [203], where they are predominantly degraded, regardless of their protease sensitivity [202].

Autophagy plays a crucial role in the degradation of protein aggregates in glia. Microglia internalize αSyn released from neurons and sequester it within autophagosomes for lysosomal degradation [173]. This process is regulated by TLR4-NF-κB signaling, which induces the expression of p62, an adaptor protein that binds to αSyn and facilitates its recruitment into autophagosomes. In the absence of TLR4, p62 upregulation is disrupted, impairing the delivery of αSyn to lysosomes in microglia [173]. Furthermore, autophagy-deficient microglia fail to clear αSyn, resulting in its accumulation and subsequent neuronal death [173]. Thus, microglial autophagy is essential for αSyn clearance and serves as a protective mechanism against neurodegeneration.

The ubiquitin-proteasome system (UPS) is also involved in the degradation of protein aggregates. In primary astrocytes, inhibition of both autophagy and the UPS significantly reduces intracellular αSyn concentrations, indicating that internalized αSyn aggregates are processed through both pathways [224]. In HD, activation of the Janus kinase/signal transducer and activator of transcription (JAK/STAT3) pathway enhances the proteolytic capacity of astrocytes by increasing the activity of lysosomal enzymes and proteasomes [225]. The JAK/STAT3 pathway is upregulated in reactive astrocytes in HD mouse and primate models, as well as in HD patients [225, 226]. Inhibition of this pathway in astrocytes of HD mouse models suppresses astrocytic reactivity but simultaneously promotes mutant HTT aggregation. Conversely, in HD mouse models with limited astrocytic reactivity, overexpression of active JAK/STAT3 in astrocytes induces astrocytic reactivity while reducing both the number and size of mutant HTT aggregates [225].

Several studies suggest that astrocytes are more efficient than neurons in the uptake and degradation of misfolded proteins, such as αSyn [227–230], TDP-43 [230] and mutant HTT [230, 231]. Astrocytes readily internalize exogenous αSyn not only from neurons but also from other astrocytes, whereas neurons exhibit a limited capacity for αSyn uptake [228]. Additionally, astrocytes efficiently degrade internalized αSyn, while in neurons, most αSyn remains intact or becomes further aggregated [227]. Furthermore, in a co-culture system of αSyn-containing neurons and naïve astrocytes, the presence of astrocytes significantly reduces αSyn fibril accumulation [228]. This difference may be attributed to the differential activity of the C-terminus of Hsp70-interacting protein (CHIP), a co-chaperone of Hsp70. CHIP is more active in facilitating the clearance of misfolded proteins in astrocytes but is inhibited in neurons [230]. These findings suggest that neurons transfer neurotoxic aggregates to astrocytes, where they are further degraded.

In disease states, however, the glial capacity to degrade protein aggregates appears to be impaired. Astrocytes derived from AD iPSCs can internalize both monomeric and aggregated tau and transport it to the lysosomal compartment [232]. However, while the uptake capacity of iPSC-derived astrocytes is comparable between control and AD groups, astrocytes from AD iPSCs exhibit defective degradation of the engulfed tau aggregates [232]. This observation likely reflects an intrinsic defect in the degradation machinery of AD astrocytes.

The phagocytic and protein degradation abilities of both microglia [233, 234] and astrocytes [66, 235] decline following Aβ uptake. Internalized Aβ prevents the translocation of TFEB to the nucleus [233], which may explain the reduced levels of the TFEB nuclear fraction observed with AD progression in patient brains [236] and the Aβ plaque-associated downregulation of endocytosis and lysosomal proteins in AD mouse models [234]. Additionally, prolonged exposure to Aβ fibrils induces lysosomal dysfunction and impairs autophagic flux in microglia [237]. Chronic activation of the C3/CR3 axis, potentially driven by Aβ, has been shown to paradoxically reduce microglial phagocytosis [102]. Thus, prolonged exposure to Aβ in human AD pathology may further compromise the ability of microglia to clear Aβ.

Pathological tau also disrupts degradation pathways in glia. Full-length tau can be degraded by the 20S proteasome into aggregation-resistant peptides [238, 239]. However, phosphorylation of tau in the microtubule-binding domain inhibits its degradation by the 20S proteasome [239]. Furthermore, the internalized Aβ [233, 235] and tau [240] induce lysosomal deacidification in astrocytes and microglia, potentially due to lysosomal membrane damage. The endosomal sorting complexes required for transport (ESCRT) machinery, which is critical for membrane repair, is recruited to lysosomes following tau internalization [240]. Inhibition of ESCRT function results in the permeabilization of tau-containing lysosomes and the escape of tau aggregates into the cytosol [240]. These findings suggest that protein aggregates may contribute to lysosomal dysfunction by creating pores in the lysosomal membrane (Fig. 2).

In primary astrocytes, the internalization of neuron-derived αSyn leads to the early upregulation of genes associated with TLR2 signaling, indicating that neuronal αSyn activates TLR2 signaling [181]. TLR2 expression is also elevated in pyramidal neurons, astrocytes, and microglia in the brains of patients with PD and DLB, as well as in αSyn-overexpressing transgenic mouse models [167, 168, 184]. TLR2 overexpression and activation exacerbate αSyn deposition in neurons and astrocytes [168, 184, 241], suggesting that exposure to pathogenic αSyn released from neurons may impair autophagy in astrocytes [242]. Similarly, under oxidative stress—a condition commonly associated with chronic disease progression and aging—oligodendrocytes also accumulate αSyn internalized from the extracellular environment [189].

It is important to note that the decreased degradation of protein aggregates can result in their release from glia (Fig. 2). Microglia have been suggested to paradoxically facilitate Aβ seeding and plaque formation, potentially by transporting and re-releasing internalized Aβ [79, 109]. Microvesicles isolated from the culture media of astrocyte and microglia co-cultures treated with Aβ were found to contain truncated forms of Aβ [243]. Similarly, after phagocytosing human tau aggregate-containing neurons, mouse primary microglia release human tau with seeding capability [135]. Tau-containing primary microglia isolated from human AD patients and tauopathy mouse models also secrete engulfed tau while retaining its seeding activity [126]. Likewise, astrocytes can secrete ingested αSyn into the conditioned medium [244].

The released proteins can contribute to pathological protein propagation and disease progression. In Drosophila, the transmission of mutant HTT from presynaptic to postsynaptic neurons is facilitated by glial phagocytosis, regulated by the phagocytic receptor Draper [245]. Mutant HTT expressed in presynaptic neurons propagates through synaptic connections and aggregates with WT HTT expressed by postsynaptic neurons, but only after first forming aggregates with WT HTT in glia [245]. The appearance of aggregates in glia precedes their emergence in postsynaptic neurons, and this progression is abolished in Draper KO models [245].

Disruption of glial lysosome acidification and degradative capacity may underlie the propagation of HTT aggregates through glia. Following Draper-dependent phagocytosis, both neuronal WT and mutant HTT localize to low-pH phagosomes and lysosomes in glia [217]. While compartments containing WT HTT maintain proper acidic conditions, those containing mutant HTT aggregates fail to sustain efficient acidification and lysosomal degradation [217]. This impaired lysosomal function in glia promotes the formation of seed-competent mutant HTT aggregates [217].

Previous studies have demonstrated that microglia, particularly DAM surrounding Aβ plaques, play a critical role in tau propagation by releasing phagocytosed tau through EVs [246]. In an Aβ-associated AD mouse model, DAM exhibit significantly upregulated expression of genes related to EV generation and secretion, accompanied by enhanced EV release [55]. Microglia-derived EVs containing pathological tau have been observed both in vitro and in vivo [246]. These EVs facilitate tau uptake by neurons in vitro and promote tau transmission to adjacent brain regions in vivo. Supporting these findings, in vitro experiments demonstrate that inhibiting EV biosynthesis reduces EV-mediated tau propagation to neurons [246].

Microglial EVs also contribute to αSyn transmission. When exposed to αSyn preformed fibrils, microglia release EVs containing internalized αSyn [247]. These αSyn-laden EVs, when taken up by recipient neurons, induce αSyn accumulation, leading to dopaminergic neuron loss and motor deficits [247]. The elevated secretion of EVs from microglia results from impaired autophagic flux [247]. Specifically, αSyn preformed fibrils upregulate E3 ubiquitin ligase expression in microglia, inducing lysosome-associated membrane protein 2 (LAMP2) degradation via the UPS [247]. Reduced LAMP2 disrupts lysosomal function and decreases autophagosome-lysosome fusion, leading to autophagosome accumulation. These autophagosomes fuse with multivesicular bodies, which release EVs upon fusion with the plasma membrane. Consequently, this autophagy blockade significantly increases the secretion of αSyn-containing EVs from microglia [247]. Similarly, astrocytes release EVs containing αSyn following lysosomal dysfunction and autophagosome accumulation after αSyn exposure [248]. These findings suggest that defects in autophagy-related proteolysis play a key role in the EV-mediated propagation of aggregated proteins.

Chaperone-mediated autophagy (CMA), a specific type of autophagy, is also implicated in the re-release of αSyn by astrocytes. In a co-culture system of neurons and astrocytes derived from human iPSCs, PD iPSC-derived astrocytes secrete and transfer αSyn to age-matched control iPSC-derived neurons, inducing neurodegenerative phenotypes [183]. This is attributed to defective αSyn degradation in astrocytes, caused by impaired lysosomal proteolysis linked to inactive CMA [183]. Notably, treatment with a CMA-activating drug rescues αSyn accumulation in PD astrocytes and co-cultured neurons, effectively preventing neuronal degeneration [183].

After actively engulfing PrP^Sc^ aggregates, astrocytes extensively transfer PrP to neurons and other astrocytes [204]. This transfer primarily occurs through the extracellular secretion of PrP from astrocytes, independent of cell death. Additionally, PrP-containing endolysosomal vesicles can be delivered via cell-to-cell connections resembling tunneling nanotubes [204]. These physical contacts significantly enhance the efficiency of PrP transmission between primary cells.

Taken together, these results suggest that the partial degradation of aggregated proteins after phagocytosis by glia may contribute to pathological protein propagation and disease progression through various secretory mechanisms.

## Therapeutic implications

The phagocytic role of glia is increasingly recognized as a promising target for therapeutic intervention. Enhancing or modulating glial phagocytic functions presents potential strategies to mitigate disease progression by reducing pathological protein accumulation and neuroinflammation.

**Fig. 3:**
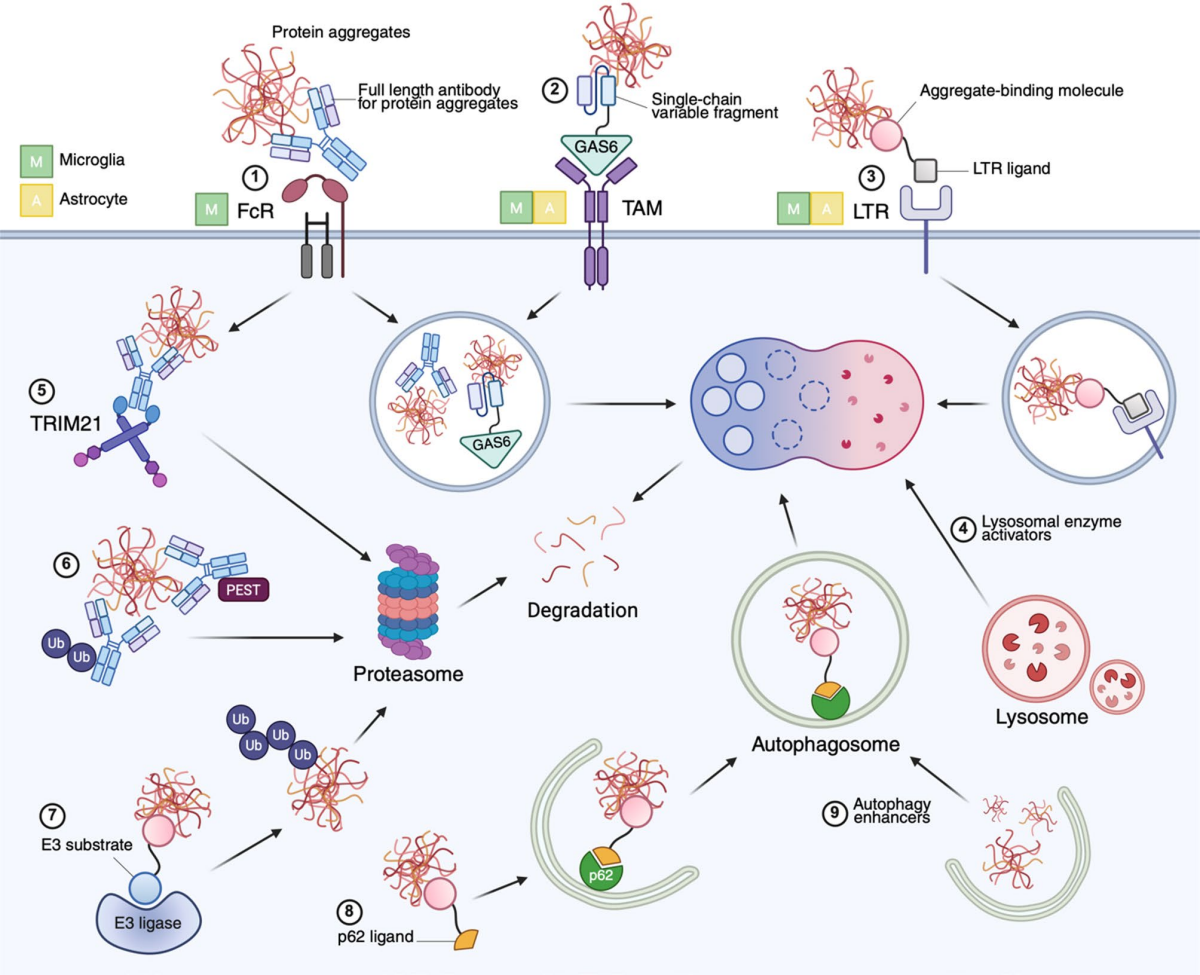
Potential therapeutic interventions targeting glial phagocytosis and the clearance of pathogenic protein aggregates. (**1**) Antibodies targeting protein aggregates or antibody-protein complexes can be internalized by Fc receptors (FcR). (**2**) Fusion proteins consisting of a single-chain variable fragment of an antibody and a ligand for TAM receptors (TAM), such as GAS6, facilitate the phagocytosis of pathogenic proteins by glia without triggering inflammatory responses. (**3**) Lysosome-targeting receptors (LTR) mediate the trafficking of plasma membrane-associated or secreted proteins to lysosomes via ligand-receptor interactions. Once internalized, aggregates are transported to lysosomes for enzymatic degradation, with (**4**) lysosomal enzyme activators enhancing this process. Within the cells, protein aggregates may be degraded via proteasomal pathways, supported by mechanisms such as (**5**) TRIM21 and (**6**) PEST motifs, or through (**7**) E3 ligase-mediated ubiquitination. Autophagy pathways also play a critical role, with (**8**) p62 ligands guiding aggregates to autophagosomes for subsequent lysosomal degradation. (**9**) Autophagy enhancers can further facilitate this pathway, promoting the clearance of protein aggregates. Cell-type specificity for each receptor is denoted by colored icon labels: green M for microglia, yellow A for astrocyte. FcR is microglia-specific, while TAM and LTR are associated with both microglia and astrocytes

For example, enhancing the phagocytic capacity of microglia is an emerging therapeutic target in immunotherapy against NDs (Fig. 3). In passive immunotherapy, antibodies work extracellularly to sequester pathological proteins and promote their clearance via microglial Fc receptor-dependent phagocytosis. Previous studies showed that anti-Aβ antibody treatments facilitate amyloid plaque clearance and improve memory deficits in AD mouse models [249–251], triggering considerable efforts to develop anti-Aβ immunotherapies for AD patients. Despite numerous clinical failures and setbacks, several anti-Aβ antibodies, including aducanumab, lecanemab, and donanemab, have demonstrated efficacy in clinical trials. Aducanumab, which received the first accelerated approval from The United States Food and Drug Administration (FDA), showed limited efficacy in cognitive improvement compared to lecanemab, the first fully FDA-approved drug [252, 253]. This difference may arise from their different binding affinities to specific Aβ species: lecanemab has a stronger affinity for protofibrils, whereas aducanumab preferentially binds fibrils [254]. Since protofibrils and oligomers are considered more neurotoxic and central to AD progression, lecanemab’s ability to target these Aβ species may explain its greater clinical effectiveness.

While passive immunotherapy has demonstrated benefits, safety concerns remain, particularly regarding amyloid-related imaging abnormalities (ARIA), the most common adverse event of antibody treatments. Although the precise mechanisms of ARIA are not fully understood, proinflammatory responses through microglia and perivascular macrophages have been implicated, potentially triggered by Fc receptor activation during anti-Aβ antibody therapy [251, 255]. To address this issue, efforts have focused on reducing Fc receptor-mediated inflammation while preserving Aβ clearance efficacy. One promising strategy involves a novel chimeric fusion protein capable of significantly eliminating Aβ plaques without inducing inflammatory responses in glia (Fig. 3) [84]. This chimeric protein replaces the PS-recognizing domain of GAS6 with the single-chain fragment variable of aducanumab, enabling Aβ clearance through TAM receptor signaling, which promotes anti-inflammatory responses. Unlike aducanumab, this approach also engages astrocytes in Aβ phagocytosis, extending therapeutic benefits beyond microglia [84]. In addition, genetic factors such as the *APOE* ε4 allele significantly increase the risk of ARIA. Clinical trials have consistently reported a higher incidence of ARIA in *APOE* ε4 homozygotes compared to heterozygotes or non-carriers [256]. These findings underscore the importance of personalizing immunotherapy based on individual genetic and biological profiles to optimize safety and efficacy.

Tau-targeted passive immunotherapy is a promising yet nascent strategy for AD intervention. Based on the assumption that tau released from neurons into the extracellular space contributes to the propagation of tau pathology, antibodies designed to sequester extracellular tau and inhibit its spread to other cells have been proposed as therapeutic interventions [257–259]. However, the efficacy of targeting extracellular tau can be limited, as the majority of tau resides intracellularly in AD [151].

Interestingly, research has shown that anti-tau antibodies or antibody-tau complexes are internalized in an Fc-dependent manner not only by microglia [125, 260], but also by neurons [261, 262]. Once internalized, antibodies bind intracellular tau and facilitate its degradation (Fig. 3). This process involves the cytosolic antibody receptor and E3 ubiquitin ligase TRIM21, which mediates the proteasomal degradation of antibody-bound tau [262]. Importantly, the protective effects of antibodies against tau pathology were reduced in the absence of TRIM21 [262], highlighting the critical role of intracellular tau clearance in the success of passive immunotherapy. Along with these passive immunotherapies, antisense oligonucleotides (ASO) targeting tau mRNA have recently been shown to reduce tau expression and ameliorate tau-related pathologies in both rodent and nonhuman primate brains [263]. Together, these data further emphasize that effective therapeutics against tauopathy may need to address both extracellular and intracellular tau species to comprehensively reduce pathogenic tau levels.

Since the incomplete degradation of pathogenic proteins engulfed by phagocytic glia may lead to their re-release, contributing to disease progression, promoting their degradation in glial lysosomes is essential to prevent the accumulation and/or re-release of toxic protein aggregates. Targeting lysosomal activity presents a direct approach to enhancing the clearance of toxic proteins. For example, ambroxol hydrochloride, a pharmacological chaperone commonly used to treat lysosomal storage disorders, increases the activity of a mutant lysosomal enzyme and has been shown to improve movement disorders in PD patients [264, 265]. Additionally, lysosome-targeting chimeras, which consist of a target-binding molecule and a lysosome-targeting ligand, can facilitate lysosomal degradation of target proteins by binding to and transporting plasma membrane-associated or secreted proteins to lysosomes [266, 267].

Pathogenic protein degradation can be enhanced by modulating autophagic flux (Fig. 3). For instance, NPT520-34, a small molecule antagonist of TLR2, enhances autophagy impaired by neuropathic proteins in PD, facilitating their clearance while reducing neuroinflammation mediated by microglia and astrocytes [268]. Similarly, dynasore, a dynamin inhibitor, increases autophagic flux by inhibiting mTORC1 activity and inducing the nuclear translocation of TFEB, resulting in autophagy-dependent removal of mutant HTT aggregates [269]. Tauopathy-homing nanoassembly (THN) is an innovative approach, consisting of a cerium oxide nanoparticle core that activates the autophagy pathway, enveloped by an antibody targeting hyperphosphorylated tau [270]. In cells containing insoluble tau aggregates, THN activates TFEB and upregulates autophagic flux, effectively eliminating pathogenic tau deposits [270]. In an AD rat model, intracerebroventricular injection of THN significantly reduces microglial activation and rescues cognitive function [270]. Autophagy-targeting chimera (AUTOTAC) is another promising therapeutic strategy that employs autophagy-dependent degradation to target specific proteins [271, 272]. AUTOTAC consists of a target-binding region linked to autophagy-targeting ligands, which interact with p62, a selective autophagy receptor. This interaction facilitates macroautophagy of the target protein. In tauopathy and PD mouse models, AUTOTAC effectively removes aggregated tau [271] and αSyn [272]. Notably, oral administration of an αSyn-targeting AUTOTAC reduces glial activation and improves behavioral deficits in a PD mouse model [272].

Another strategy to promote the clearance of toxic proteins involves utilizing the proteasome (Fig. 3). Anti-tau intrabodies fused to ubiquitin, which shuttle intracellular tau to the proteasome, significantly reduce tau protein levels and mitigate tauopathy [273]. Similarly, the proteasome-targeting motif PEST (proline, glutamic acid or aspartic acid, serine, and threonine) can enhance proteasome-mediated clearance. Intrabodies engineered with the PEST motif augment the degradation of HTT [274] and αSyn aggregates [275] in vitro and have demonstrated efficacy in vivo, preventing synucleinopathy in a viral αSyn overexpression model of PD [276]. Additionally, Proteolysis-targeting chimeras (PROTACs), which induce selective binding of an E3 ubiquitin ligase to a substrate protein, can facilitate targeted proteolysis [277]. A small molecule [278] or antibody [279] that recruits the protein of interest triggers its ubiquitination by the E3 ligase, leading to proteasome-mediated clearance. PROTACs designed for tau degradation have been shown to promote tau clearance via proteasome-dependent proteolysis, ameliorating tau accumulation, synaptic abnormalities, and cognitive deficits [278]. Although there have been no cases yet of directly enhancing glial degradation capacity to mitigate NDs, these approaches may become viable using the methods described above.

## Conclusion

Previous efforts to identify the mechanisms of ND progression and develop therapeutic applications have primarily focused on neuronal cells. In this review, however, we explore and emphasize the role of glia in NDs, particularly their phagocytic functions. The mechanisms by which glia internalize, process, and potentially release neurotoxic protein aggregates highlight their dual roles in both protective and detrimental effects. While various glial cells demonstrate the ability to engulf and degrade synapses and protein aggregates, their efficiency is often altered in disease states, exacerbating pathology.

Therapeutic strategies aimed at enhancing glial phagocytosis and restoring their functional capacity hold promise for the treatment of NDs. However, activating general innate and adaptive immune responses should be approached with caution since it can result in the removal of healthy synapses and neurons as well as the unintended immune responses, exacerbating neurodegeneration rather than alleviating it. For example, activating TREM2 function with agonistic antibodies can induce excessive synapse loss near Aβ plaque [280]. Moreover, Fc receptor activation during anti-Aβ antibody therapy can activate pro-inflammatory responses, enhancing neuroinflammation and smooth muscle cell loss in the vasculature [281, 282].

Therefore, future research should focus on elucidating the precise molecular pathways governing glial phagocytosis and subsequent lysosomal functions, as well as the complex interactions between glial and neuronal cells in both health and disease. Engineering chimeric molecules that specifically activate the beneficial phagocytic functions of glia represents a promising approach to treat NDs, as it allows for the artificial redirection of glial clearance functions while suppressing excessive inflammatory responses. This approach may overcome the challenge of balancing both glial activation and neuroprotection, ultimately leading to more effective and safer interventions for NDs.

## Data Availability

Data sharing not applicable to this article as no datasets were generated or analysed during the current study.

## Abbreviations

NDs: Neurodegenerative diseases
CNS: Central nervous system
AD: Alzheimer’s disease
PD: Parkinson’s disease
HD: Huntington’s disease
ALS: Amyotrophic lateral sclerosis
FTD: Frontotemporal dementia
Aβ: Amyloid beta
αSyn: Alpha-synuclein
OPCs: Oligodendrocyte precursor cells
TREM2: Triggering receptor expressed on myeloid cells 2
TLRs: Toll-like receptors
TAM: TYRO3, AXL, and MERTK receptor protein tyrosine kinases
CR3: C3 receptors
PS: Phosphatidylserine
KO: Knockout
LDAM: Lipid-droplet-accumulating microglia
APDAs: Autophagy-dysregulated astrocytes
MS: Multiple sclerosis
APOE: Apolipoprotein E
SOD1: Superoxide dismutase 1
APP: Amyloid precursor protein
DAM: Disease-associated microglia
SR-A: Scavenger receptor A
LRP1: Low-density lipoprotein receptor-related protein 1
NFTs: Neurofibrillary tangles
EC: Entorhinal cortex
EVs: Extracellular vesicles
CR4: Complement receptor 4
HSPGs: Heparan sulfate proteoglycans
DLB: Dementia with Lewy bodies
MSA: Multiple system atrophy
RAGE: Receptor for advanced glycation end products
PrP: Prion protein
HTT: Huntingtin protein
TFEB: Transcription factor EB
UPS: Ubiquitin-proteasome system
JAK/STAT3: Janus kinase/signal transducer and activator of transcription
CHIP: C-terminus of Hsp70-interacting protein
ESCRT: Endosomal sorting complexes required for transport
LAMP2: Lysosome-associated membrane protein 2
CMA: Chaperone-mediated autophagy
FDA: The United States Food and Drug Administration
ARIA: Amyloid-related imaging abnormalities
THN: Tauopathy-homing nanoassembly
AUTOTAC: Autophagy-targeting chimera
PEST: proline, glutamic acid or aspartic acid, serine, and threonine
PROTACs: Proteolysis-targeting chimeras
